# Age-Related Changes in Trabecular and Cortical Bone Microstructure

**DOI:** 10.1155/2013/213234

**Published:** 2013-03-18

**Authors:** Huayue Chen, Xiangrong Zhou, Hiroshi Fujita, Minoru Onozuka, Kin-Ya Kubo

**Affiliations:** ^1^Department of Anatomy, Gifu University Graduate School of Medicine, 1-1 Yanagido, Gifu 501-1194, Japan; ^2^Department of Intelligent Image Information, Gifu University Graduate School of Medicine, 1-1 Yanagido, Gifu 501-1194, Japan; ^3^Nittai Jusei Medical College for Judo Therapeutics, 2-2-7 Yoga, Setagaya-ku, Tokyo 158-0097, Japan; ^4^Seijoh University Graduate School of Health Care Studies, 2-172 Fukinodai, Tokai, Aichi 476-8588, Japan

## Abstract

The elderly population has substantially increased worldwide. Aging is a complex process, and the effects of aging are myriad and insidious, leading to progressive deterioration of various organs, including the skeleton. Age-related bone loss and resultant osteoporosis in the elderly population increase the risk for fractures and morbidity. Osteoporosis is one of the most common conditions associated with aging, and age is an independent risk factor for osteoporotic fractures. With the development of noninvasive imaging techniques such as computed tomography (CT), micro-CT, and high resolution peripheral quantitative CT (HR-pQCT), imaging of the bone architecture provides important information about age-related changes in bone microstructure and estimates of bone strength. In the past two decades, studies of human specimens using imaging techniques have revealed decreased bone strength in older adults compared with younger adults. The present paper addresses recently studied age-related changes in trabecular and cortical bone microstructure based primarily on HR-pQCT and micro-CT. We specifically focus on the three-dimensional microstructure of the vertebrae, femoral neck, and distal radius, which are common osteoporotic fracture sites.

## 1. Introduction

The proportion of elderly persons in the world population has increased substantially and will continue to do so in the coming years. Aging has multiple complex effects that result in the progressive deterioration of various organs, including the skeleton. Age-related bone loss and osteoporosis in the elderly increase the risk of fractures and morbidity in this population [1]. Osteoporosis is a common disease of the elderly [2–4], characterized by low bone mass and microstructural deterioration of bone tissue, with an increased fracture risk. Osteoporosis is defined by the World Health Organization as a bone mineral density (BMD) at least 2.5 standard deviations below the mean peak bone mass of young, healthy adults as measured by dual-emission X-ray absorptiometry [5]. With the aging population, osteoporosis and its related fractures have become an increasingly important health and socioeconomic issue. It is important to identify the possible pathologic mechanisms underlying bone fragility in old age. During life, mammalian bone undergoes a process of continuous remodeling in which old bone is resorbed and replaced with newly formed bone. In young adults, the overall amounts of resorbed and formed bone are balanced. With aging, however, this remodeling balance becomes negative, resulting in a decreased bone mass. The decline in bone mass is associated with reduced bone strength, resulting in osteoporosis [6, 7].

Osteoporosis is generally asymptomatic. The main consequence of osteoporosis is the increased risk of bone fractures. The vertebrae, femoral neck, and distal radius are highly susceptible to fracture in patients with osteoporosis [1–3]. Although BMD is an important predictor of subsequent fracture risk, age itself is also a major determinant factor of the fracture risk, independent of BMD [6, 7]. The effects of age on fracture risk could be due to a number of factors, including bone microstructural deterioration and possible changes in bone material properties, such as the composition and degree of collagen cross-linking. Although techniques to noninvasively measure bone material properties may be validated and available in the future, high-resolution computed-tomography (CT) currently allows for three-dimensional (3D) assessment of the bone microstructure and evaluation of the trabecular and cortical microstructure separately [8]. The present paper discusses recently studied age-related changes in trabecular and cortical bone microstructure, based primarily on high resolution peripheral quantitative CT (HR-pQCT) and micro-CT. As these changes take a somewhat different course in females and males and in different skeletal sites, these variations are also reviewed.

## 2. Vertebrae

Fractures caused by osteoporosis most often occur in the vertebrae. The vertebrae from the mid-thoracic region down are more likely to sustain osteoporosis-related fractures [9]. Osteoporosis-related vertebral fractures may occur spontaneously or during everyday activities, such as opening a window, an insignificant fall, or twisting while lifting. The vertebrae bear the weight of the upper body and withstand substantial physical pressure. The vertebral body, a box-shaped block of bone, comprises an elliptical block of trabecular bone covered by a thin shell of cortical bone. Vertebral bodies are connected to each other by intervertebral discs at the upper and lower ends and ligaments at the anterior/posterior lateral sides, forming a flexible column. This design provides a lightweight structure that involves a minimum of material in its construction. The quality of the trabecular bone in vertebral bodies plays an important role in the performance of the entire spine [10, 11].

### 2.1. Vertebral Trabecular BMD

**Figure 1:**
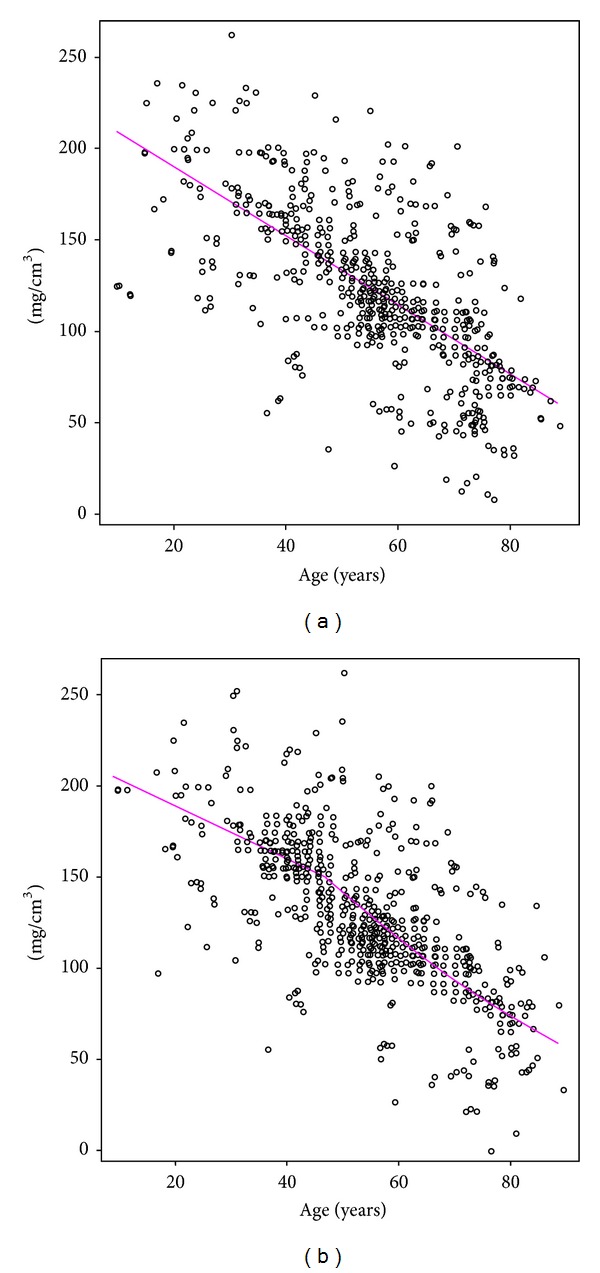
Relationship between age and trabecular vBMD at the third lumbar vertebra for men (a) and women (b) [13].

The trabecular BMD in the vertebrae is metabolically more active and may therefore serve as an early indicator of vertebral osteoporosis [8]. Vertebral trabecular BMD is significantly correlated with vertebral fracture [10]. Worldwide, the number of subjects undergoing thoracic and abdominal CT examinations has increased dramatically over the last two decades [8, 12]. In an age- and sex-stratified population sample of 541 women and 490 men aged 17 to 88 years, we examined the relationship between vertebral trabecular volumetric BMD (vBMD) and age [13]. The CT images included all thoracic and lumbar vertebrae and were scanned using standard setting (120 kV, auto mAs, 1.25 mm thick slice, pitch = 0.49–0.88 mm). Slice intervals were modified to the same value as the pitch using sinc interpolation to keep each voxel in an isotropic size in three dimensions. A standard phantom (B-MAS 200; Kyoto Kagaku, Kyoto, Japan) was used to calibrate the CT Hounsfield units to equivalent bone mineral concentration [13]. Trabecular vBMD of both women and men tended to decrease gradually from the first thoracic vertebra (Th1) to the third lumbar vertebra (L3) in all age categories. With regard to the vertebral level, L3 had the lowest vBMD among the thoracic and lumbar vertebrae. Compared with Th1, trabecular vBMD of L3 was lower than that of Th1 by about 30% [13]. Trabecular vBMD of L3 tended to decrease with aging for both women and men. The vBMD in subjects over 70 years of age was lower than that of adults under 40 years of age by about 70%. In women 50 years of age and older, the vBMD was considerably lower than that in women under 50 years of age. In men, trabecular vBMD declined at an almost constant rate with aging (Figure 1).

### 2.2. Vertebral Trabecular Microstructure

Vertebral trabecular bone has a complex inhomogeneous 3D microstructure [14, 15]. The central and anterosuperior regions of the vertebral body have a lower bone volume fraction (BV/TV) than the corresponding posterior region [14, 16]. A thorough understanding of the regional variations in microstructural properties is crucial for evaluating age- and sex-related bone loss of the vertebrae and may provide more insight into the mechanisms of vertebral osteoporosis and the related fracture risks. We studied 56 fourth lumbar vertebrae (L4) from Japanese cadaver donors aged 57 to 98 years [14]. An 8 mm thick sagittal section close to the midline of the L4 vertebral body was harvested using a water-cooled low-speed diamond saw (Buehler IsoMet, Illinois, USA). The central trabecular bone of the vertebral body was examined by micro-CT and scanning electron microscopy. The trabecular bone volume fraction (BV/TV) and trabecular number (Tb.N) significantly decreased with aging. Between the ages of 60 and 90 years, BV/TV declined by 22% and 24% in both women and men. Decreases in BV/TV with aging were similar in women and men. Tb.N also declined with aging, by 16% in women and 19% in men. Consistently, trabecular separation (Tb.Sp) increased with aging. The age-related decrease in trabecular thickness (Tb.Th) was not statistically significant [14, 15]. The reduction of BV/TB with aging is associated primarily with reduced Tb.N and increased Tb.Sp [14, 17].

Age-related changes of Tb.Th are quite controversial. Some studies report a greater relative loss and thinning with age for all trabeculae [18], or for horizontal trabeculae only [19]. With the loss of horizontal trabeculae, the remaining vertical trabeculae tend to maintain their thickness and might even increase in thickness with aging [14, 19]. Some studies indicate that there are no significant changes in Tb.Th with aging [14]. The reduced BV/TV due to decreases in Tb.N and increases in Tb.Sp, with or without thinning of Tb.Th, has formed the basis of the plausible hypothesis for age-related trabecular bone loss [20].

Some microstructural parameters differ significantly between women and men [14, 21]. Compared with women, men have higher BV/TV and Tb.N. Scanning electron microscopic images revealed increased resorbing surfaces, perforated or disconnected trabeculae, and microcallus formations in elderly subjects [14, 22, 23]. Therefore, it is conceivable that age-related vertebral trabecular bone loss is caused by increased bone resorption activity [9, 10, 14]. These findings illustrate some potential mechanisms underlying vertebral fractures.

### 2.3. Vertebral Cortical Bone

The cortical shell of the vertebral body is thin and porous. Thus, one difficulty in sorting out the role of the vertical cortex, particularly in aged individuals, is that the extreme thinness of the cortex makes it difficult to measure with most nondestructive techniques. The relative contribution of the cortical shell to whole bone strength remains poorly understood. The vertebral cortical thickness ranges from 180 to 600 *μ*m, with a mean thickness of 380 *μ*m [24–27]. The cortical thickness of the thoracic vertebrae is thinner than that of the cervical and lumbar vertebrae. The mean thickness of the ventral shell is in general greater than that of the dorsal shell. Cortical thickness is not sex specific. Cortical thickness slightly decreases with aging. Although most studies emphasize the important role of trabecular bone in age-related vertebral fragility, both old and new studies point to an important role for the cortical shell, particularly when trabecular bone volume is low, in elderly subjects [24–27].

## 3. Femoral Neck

Femoral neck fractures are the most common injury observed in elderly subjects. This type of fracture is a major cause of morbidity in the elderly as it leaves many patients immobile and confined to their bed [28]. The risk of femoral neck fracture increases 10-fold with every 20 years of age. Femoral neck fractures usually occur due to falls, which are common among the elderly. Femoral neck fractures are attributed to both cortical and trabecular bone loss. The anatomic distribution of cortical and trabecular bone in the femoral neck might be critical in determining resistance to fracture.

### 3.1. Microstructure of Femoral Neck Trabecular Bone

An approximately 15 mm segment of femoral neck was harvested by cutting at the base of femoral head and at the base of femoral neck. Trabecular specimen of 8 × 8 × 8 mm cube was obtained in the middle of femoral neck for micro-CT analysis. Age-related changes in trabecular bone of the femoral neck include a decrease in BV/TV, Tb.N, and an increase in Tb.Sp [29, 30]. BV/TV declines by 22% and 18% between ages 60 and 90 years. Tb.N and Tb.Th decrease, and Tb.Sp increases in both women and men. The reduction of BV/TV with aging is associated with a decline in Tb.N and Tb.Th and increase in Tb.Sp [21, 29–32]. Trabecular bone in the femoral neck has a complex 3D structure that consists of interconnecting plates and rods. Plate or rod characteristics of trabeculae can be estimated by measuring the structure model index (SMI). This is an important structural feature that strongly impacts the mechanical properties of trabeculae [33]. The SMI in the femoral neck and vertebrae significantly increases with aging [29, 32]. A more rod-like structure of trabecular bone is observed in the femoral neck with aging, and, hence, the femoral neck is likely to be more susceptible to bending and buckling failure modes. Trabecular connectivity is a fundamental property of 3D networks. Connectivity density (Conn.D) is crucial in the maintenance of bone strength. Conn.D in femoral neck and vertebra decreases significantly with aging [14, 29]. As the trabecular bone volume decreases, there is a corresponding decrease in Conn.D, possibly due to the loss of small interconnecting trabeculae with a small initial diameter [14]. The degree of anisotropy (DA) defines the direction and magnitude of the preferred orientation of trabeculae and uses the ratio between the maximum and minimum radii of the mean intercept length ellipsoid [34]. DA is a measure of how highly oriented substructures are within a volume, which is an important trabecular bone microstructural parameter. In the trabecular bone of femoral neck and vertebra, we did not find any significant differences between DA and age.

### 3.2. Microstructure of Femoral Neck Cortical Bone

The morphology of the femoral neck shows marked regional heterogeneity [29, 35–37]. As the body weight rests vertically and unidirectionally on the hip joint, cortical bone in the superior region is thinner than that in the inferior region of the femoral neck. The cortices in the elderly exhibit marked thinning in the superior region, but the inferior cortices are thicker compared with those in younger adults [35–37]. Relative to the mean value at age 60 years, cortical thickness in the superoposterior octant, which is compressed most in a sideways fall, declines in women by 6.4% per decade. Similar but significantly smaller effects are evident in men. This thinning compromises the capacity of the femoral neck to absorb energy independently of osteoporosis [29, 36].

**Figure 2:**
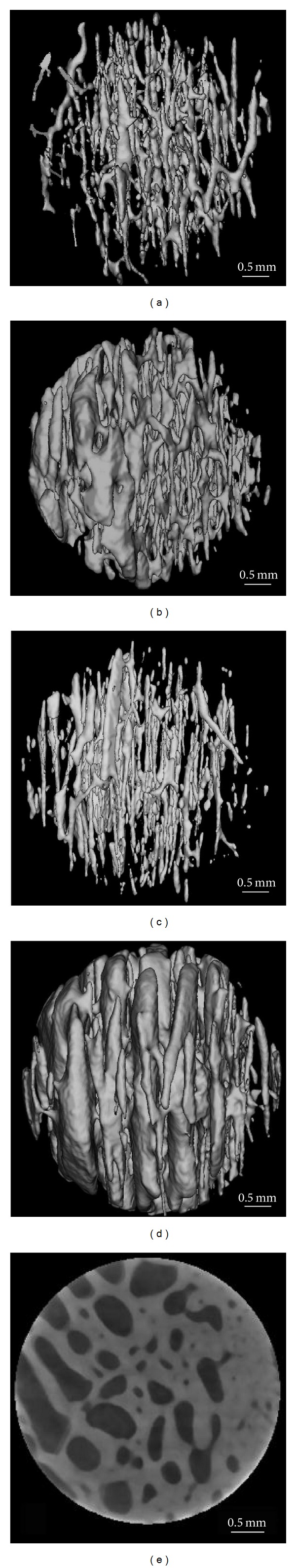
3D reconstructed images of cortical porosity at the inferior femoral neck from a man aged 62 years (a), a man aged 92 years (b), a woman aged 62 years (c), and a woman aged 92 years (d). There are more enlarged pores in the 92-year-old group than those of the 62-year-old group. Representative 2D micro-CT image of the femoral neck cortex from a woman aged 92 years (e) is shown. The periosteal surface faces right for all specimens [29].

The cortical porosity (Ct.Po) of the femoral neck ranges from 5% to 13% [29, 36–38]. With aging, pores within the cortex adjacent to the marrow cavity coalesce, leaving cortical remnants that look similar to trabeculae. The remaining thinned cortex beneath the periosteum retains a compact appearance and contains enlarged though not confluent pores. In elderly subjects, excavation of the remaining compact cortex leaves further cortical remnants.  Figure 2 shows the age-related changes in cortical porosity of the femoral neck. Between ages 60 and 90 years, cortical thickness (Ct.Th) decreases by 3% to 5% per decade, and Ct.Po increases by 31% to 33% per decade [29]. The pore diameter increases, with no significant changes in the pore number [29, 36–38]. Consequently, we consider that cortical porosity with aging is mainly due to enlarged intracortical pores. Relative to men, women have a higher Ct.Po and pore diameter. Therefore, while age is the most important factor, sex also has a role in Ct.Po and pore size. With aging, cortical pores fuse together to form giant pores with diameters exceeding 385 *μ*m [29, 36, 39]. Thus, the formation of giant pores could be considered a pivotal process in the focal loss of cortical thickness and strength.

The most obvious age-related change in femoral neck is the increase in Ct.Po. The decrease of BV/TV with aging is more noticeable than that of Ct.Th. There is a significant inverse correlation between Ct.Po and BV/TV for both women and men. As compared with women, men have higher Ct.Th and BV/TV and lower Ct.Po. These findings may serve as reference for ethnic comparison with aging and sex and may provide more insight into femoral neck fracture risk [29, 36, 39].

## 4. Radius

Fractures of the distal radius are one of the most common injury types, especially in pediatric and elderly populations, which are at greatest risk for this injury [40]. As the population continues to age, the incidence of osteoporotic distal radius fractures will also increase. In the elderly population, radial fractures frequently result from falls from a standing height and other low-energy traumas [41].

Population studies using HR-pQCT imaging revealed that in the trabecular bone of the distal radius, between the ages of 20 and 90 years, BV/TV decreases by 27% in women and 26% in men. BV/TV remains relatively constant at the distal radius until midlife and declines thereafter. Tb.N declines and Tb.Sp increases with aging in women. Trabecular bone mass is higher in men than in women of the same age; age-related declines of the trabecular BMD and BV/TV are similar in women and men between the ages of 20 and 90 years [42–45]. The microstructural basis for the decrease in trabecular volume differs between women and men [44, 45]. Women appear to lose trabeculae, primarily with reductions in Tb.N and increases in Tb.Sp, while in men the main mechanism for the decrease in BV/TV is trabecular thinning, resulting in a marked decrease of Tb.Th and unchanged Tb.N [44, 45].

Cortical bone was examined morphologically with HR-pQCT. Cortical bone strength (failure load) and the load distribution were estimated using finite-element analysis as reported previously [46]. Cortical vBMD is significantly lower in older women than in younger women. There is no significant change in the cortical vBMD with age in men [44]. Ct.Po is very low in the radius, ranging from 0.2% to 2.4% [44, 45]. Older women and men have an increased Ct.Po and cortical pore diameter, compared with younger subjects. Cortical bone strength correlates negatively with Ct.Po, which is an important component of bone quality that deteriorates with aging, independent of BMD [45, 47, 48]. The age-related increase in Ct.Po is more than 2-fold greater in women than in men. Cortical thickness also tends to decline more with aging in women than in men. Bone strength is greater in men than in women at the distal radius. The sex difference is probably caused by greater cortical porosity in women.

## 5. Tibia

Although the tibia is commonly measured with pQCT, there are currently no recommendations for using the tibia for bone health assessment or hip fracture risk prediction. Given that the tibiae are exposed to multiple modes, frequencies, durations, amplitudes, and rates of mechanical loading from physical activities, tibial characteristics may be more closely related to those of the hip than the forearm [49, 50].

**Figure 3:**
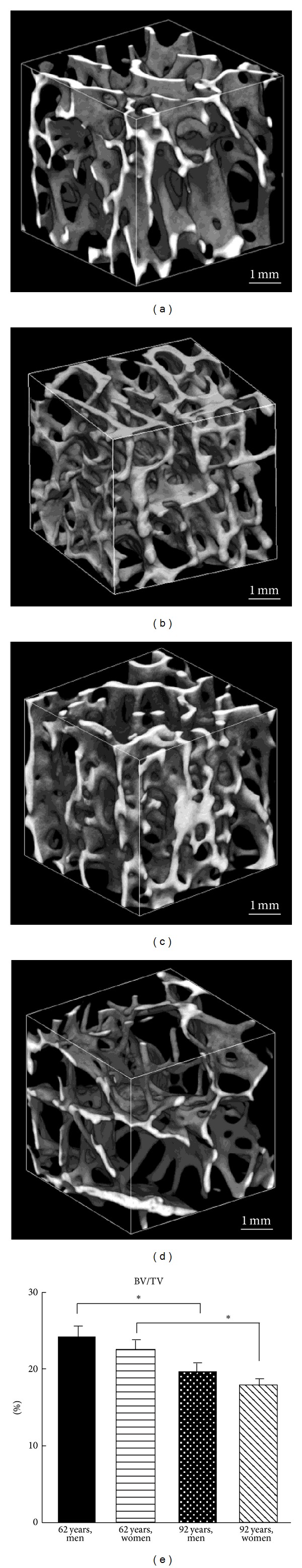
3D reconstructed images of trabecular microstructure at the proximal tibia from a man aged 62 years (a), a man aged 92 years (b), a woman aged 62 years (c), a woman aged 92 years (d), and the corresponding values for BV/TV (e). The trabecular bone volume fraction is highest in man aged 62 years and lowest in woman aged 92 years (*P < 0.05) [51].

Trabecular bone specimens from the medial compartment of the proximal tibial metaphyses have been examined with micro-CT and scanning electron microscopy [51–53]. Trabecular BMD and BV/TV of the proximal tibia show age-related decreases in women and men [51–53].  Figure 3 shows the age-related changes of trabecular bone at the proximal tibia. From 57 to 98 years of age, BV/TV decreases by 7% and 6% per decade for women and men, respectively, while BMD declines by around 4% per decade. The rate of decline with aging is similar for women and men. Women, however, have a consistently lower BMD and BV/TV than men of the same age. Possible explanations for the sex differences are that women reach a lower peak bone mass before they start losing bone, or that any accelerated loss occurs earlier, perhaps, as has been suggested, around menopause [51, 54]. It is likely that much of the female preponderance for fractures is related to the lower bone mass of women compared with men.

Age-related trabecular microstructural changes at the proximal tibia include a decrease in BV/TV, Tb.Th, and Conn.D, as well as an increase in Tb.Sp and SMI [51–53]. The decline in BV/TV and Tb.Th with aging is similar for women and men. The age-related decrease in Tb.N for women is nearly twice that in men. Age-related bone loss at the proximal tibia in women is considered to be due to decreases in both Tb.N and Tb.Th, whereas in men, the primary mechanism for the decrease in BV/TV is trabecular thinning. Based on finite element modeling, reductions in Tb.N have a 2- to 5-fold greater impact on bone strength compared with reductions in Tb.Th that result in similar decreases in bone volume [55]. The SMI increases with aging. A shift toward a more rod-like structure with aging is observed in the proximal tibia [51, 53].

Scanning electron microscopy revealed that the percentage area of trabecular resorbing surface increases significantly with aging. Some trabeculae are completely perforated or disconnected. Age-related trabecular bone loss at the proximal tibia is caused by trabecular perforation and thinning. Several trabecular microcallus formations on the thin trabeculae are observed in elderly subjects. A microcallus is a small mass of woven bone often observed at the vertebra, mainly on the vertical trabeculae [14, 23, 51]. A microcallus can be seen as an attempt to preserve or repair a trabecula [14, 56, 57]. What triggers the microcallus formation, however, remains a subject of debate.

Cortical thickness of tibia tends to decline more with aging in women than in men. Ct.Po in the tibia ranges from 0.3% to 7.1%, which is lower than that of the femur and higher than that of the radius [44, 45, 48, 58]. Men have a larger cortical area and thicker cortices than women. Older women and men have an increased Ct.Po and cortical pore diameter compared with younger subjects. The age-related increase in Ct.Po is more prominent in women. The sex difference in tibial strength is due to greater trabecular bone mass and lower cortical porosity.

## 6. Conclusion

Age-related bone loss and resultant osteoporosis in a substantial proportion of the elderly population is multifaceted and multifactorial, involving a progressive loss of both bone quantity and quality. A number of genetic, hormonal, and biochemical players are implicated in this process. The role of aging pathways, such as increased oxidative stress and telomere shortening, in bone loss is also becoming apparent. The role of nutrition and lifestyle choices such as exercise is better appreciated with newer studies linking decreased bone density and increased fracture risk in nutritionally compromised and sedentary elderly individuals.

Age-related microstructural changes in bone are complex. There are three major age-related processes that lead to bone loss. The first and most important is trabecular bone loss. The decrease in trabecular bone is caused by thinning of the trabeculae and, especially in early postmenopausal women, by disruption of the trabecular microstructure and loss of trabecular elements. Trabecular bone loss over life is one-half at the vertebra and one-quarter at the femur, radius, and tibia. The second process contributing to bone loss is a decrease in cortical bone, mainly caused by increased porosity from both an increase in resorption cavities and an accumulation of incompletely closed osteons with aging. The third process is continued net resorption at the endocortical surface. Bone loss over life from this process is approximately 25% to 40% at the femoral neck and distal radius, but less at the tibia.

Vertebral strength, a key etiologic factor of osteoporotic fractures, is maintained mainly by trabecular bone. Vertebral trabecular bone mass is lower than peripheral bone mass. Vertebral trabeculae are microstructurally heterogeneous, with lower bone mass at the central and anterosuperior regions of the vertebral body. Decreased trabecular bone mass and especially increased cortical porosity might be the most important causes of femoral neck and tibial fragility in the elderly subjects. Cortical porosity is an important component of bone quality at the distal radius that deteriorates with aging. Although our understanding of the pathogenesis of aging bone is appreciable, it is not yet exhaustive. Further studies are needed to define the extent to which deterioration of the cortical and trabecular microstructure contributes to the effect of age on bone fragility at common sites of osteoporotic fractures.

## Abbreviations

BMD: Bone mineral density
BV/TV: Bone volume fraction
Tb.Th: Trabecular thickness
Tb.N: Trabecular number
Tb.Sp: Trabecular separation
Ct.Po: Cortical porosity
SMI: Structural model index
Conn.D: Connectivity density
DA: Degree of anisotropy
vBMD: Volumetric bone mineral density
CT: Computed tomography
HR-pQCT: High resolution peripheral quantitative CT.
